# Beyond Squamous Cell Carcinoma: Basaloid Squamous Cell Carcinoma of the Esophagus

**DOI:** 10.7759/cureus.12619

**Published:** 2021-01-11

**Authors:** Heeyah Song, Eula Tetangco, Loc Ton, Amanda Barrett, John Erikson L Yap

**Affiliations:** 1 Medicine, Medical College of Georgia at Augusta University, Augusta, USA; 2 Gastroenterology and Hepatology, Medical College of Georgia at Augusta University, Augusta, USA; 3 Gastroenterology, The Permante Medical Group, Sacramento, USA; 4 Pathology, Medical College of Georgia at Augusta University, Augusta, USA

**Keywords:** ec- esophageal cancer, basaloid squamous cell carcinoma, esophageal basaloid squamous cell carcinoma, bscc

## Abstract

Basaloid squamous cell carcinoma (BSCC) is a poorly differentiated variant of squamous cell carcinoma (SCC) with distinct morphologic characteristics. Yet, there are no clearly defined guidelines established for management. BSCC in the esophagus is a very rare entity, with the proportion of esophageal BSCC ranging from 0.068% to 11%. This wide range is thought to be secondary to difficulty making the diagnosis on small biopsy specimens and the lack of a universally defined proportion of BSCC components necessary to make the diagnosis. We present the case of a 57-year-old African American female, who underwent esophagogastroduodenoscopy (EGD) after an abnormal barium swallow in the setting of two months history of dysphagia and weight loss and was diagnosed with BSCC of the esophagus on histopathology.

## Introduction

This article was previously presented as a meeting abstract at the 2020 American College of Gastroenterology Annual Scientific Meeting Virtual in October 2020. 

Esophageal cancer is the sixth leading cause of cancer-related death in the world with five-year survival around 15-20% [1]. Esophageal squamous cell carcinoma (SCC) is the most common histological type worldwide, but in Western Europe and the United States, the adenocarcinoma subtype is the predominant type [1]. Basaloid squamous cell carcinoma (BSCC) is a poorly differentiated variant of SCC that frequently has both basaloid and conventional SCC components [2]. This morphologic variant was first described in the upper aerodigestive tract by Wain et al. in 1986 as a tumor composed of closely packed cells with hyperchromatic nuclei and scant cytoplasm. BSCC is a tumor that is thought to originate from totipotent cells in the basal layer of the squamous epithelium, thus giving rise to this characteristic appearance [3,4]. BSCC occurs more commonly in the head and neck, especially in the supraglottic larynx, tongue, and hypopharynx, than in the digestive tract. But even in the larynx, the SCC basaloid subtype accounts for less than 1% of laryngeal carcinoma [5]. In the esophagus, it represents only 0.068-11.0% of all esophageal carcinomas [6]. Given the rarity of esophageal BSCC, there are no clear differentiating guidelines for its management [7]. We present the rare case of a 57-year-old female referred for endoscopic evaluation for dysphagia who was found to have BSCC of the esophagus.

## Case presentation

**Figure 1:**
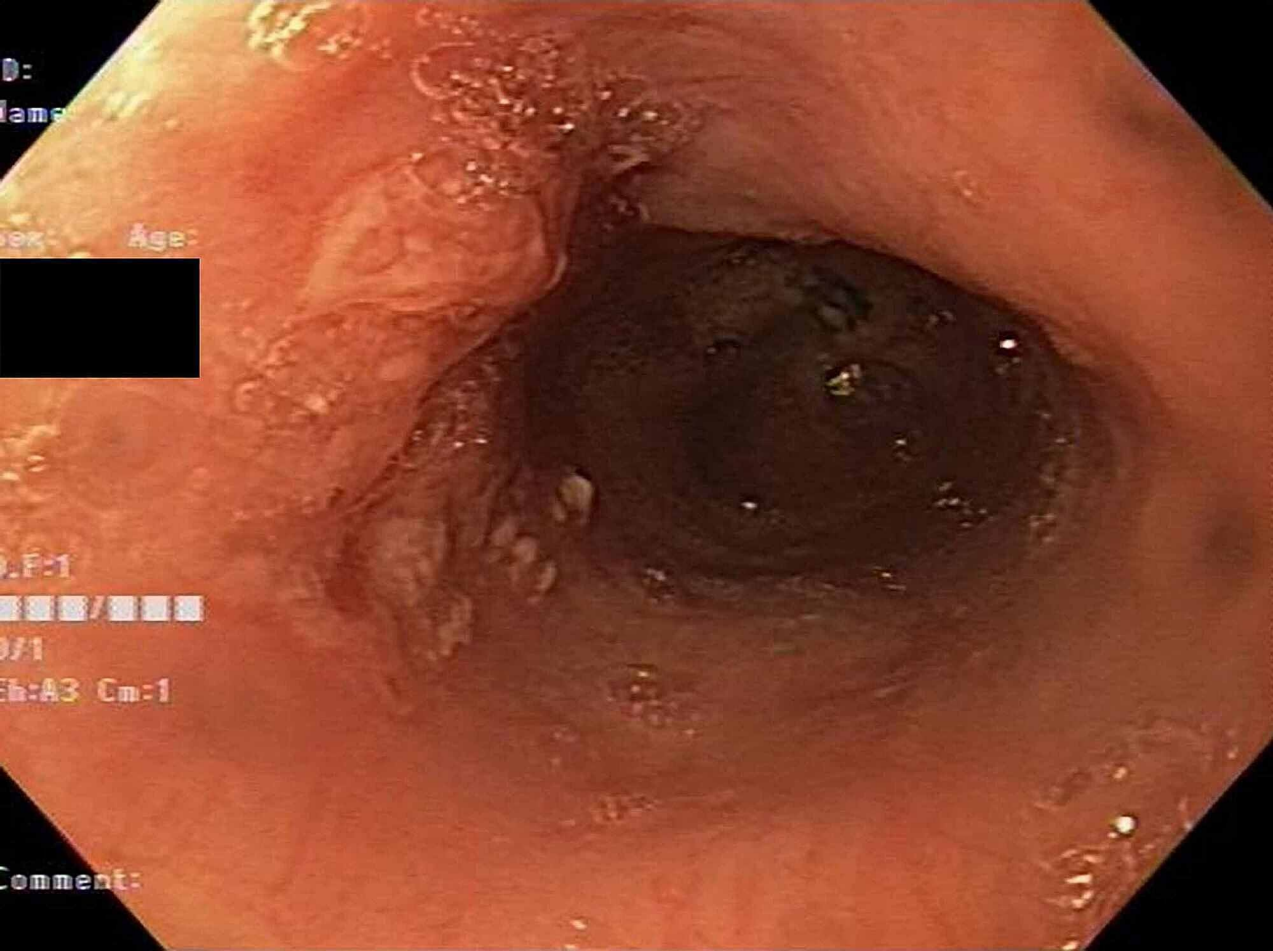
EGD shows nodular mucosa with ulceration at 23 cm from the incisors EGD: esophagogastroduodenoscopy.

**Figure 2:**
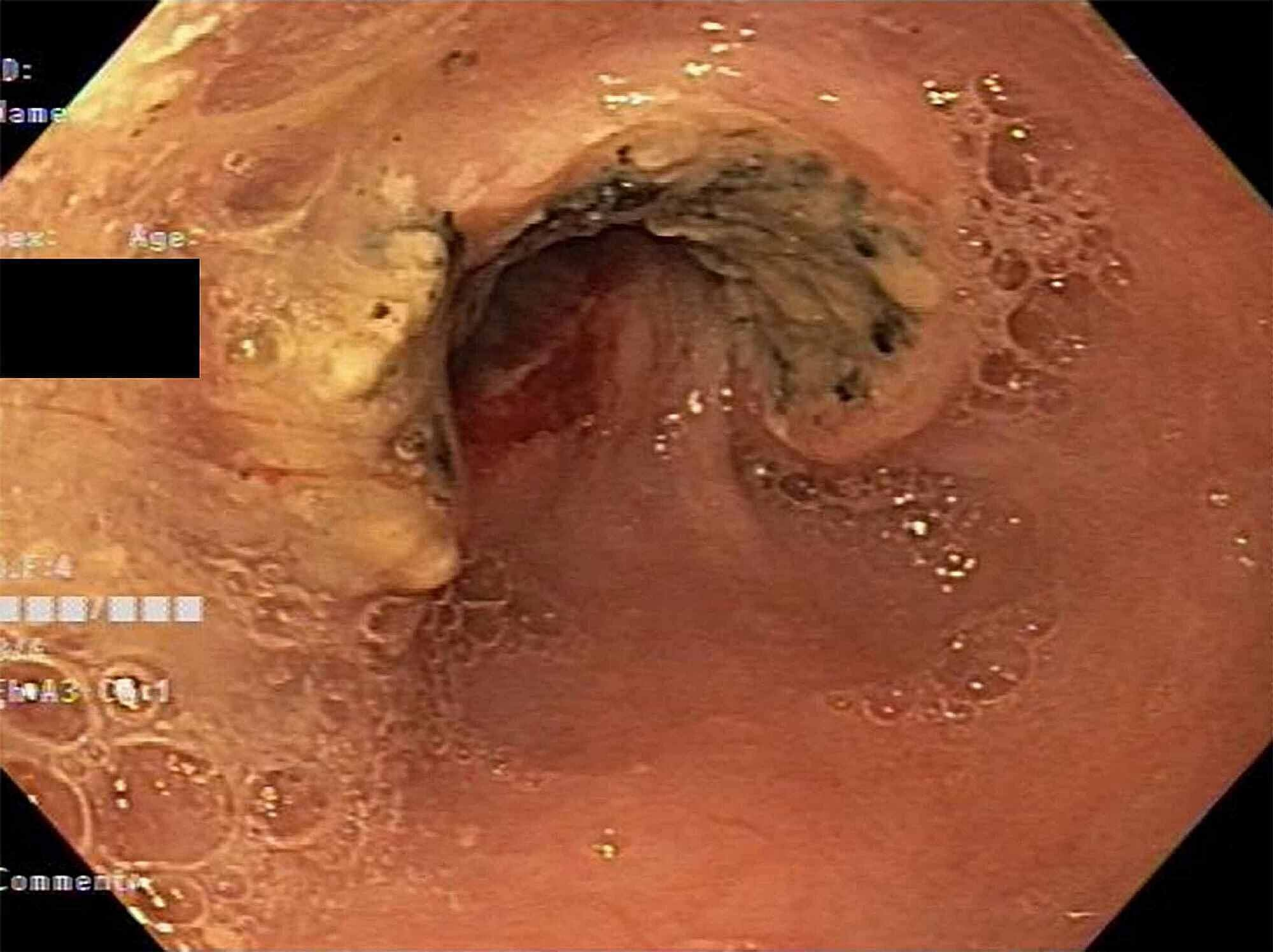
EGD shows deeply invasive, large bulky ulcerated fungating masses EGD: esophagogastroduodenoscopy.

**Figure 3:**
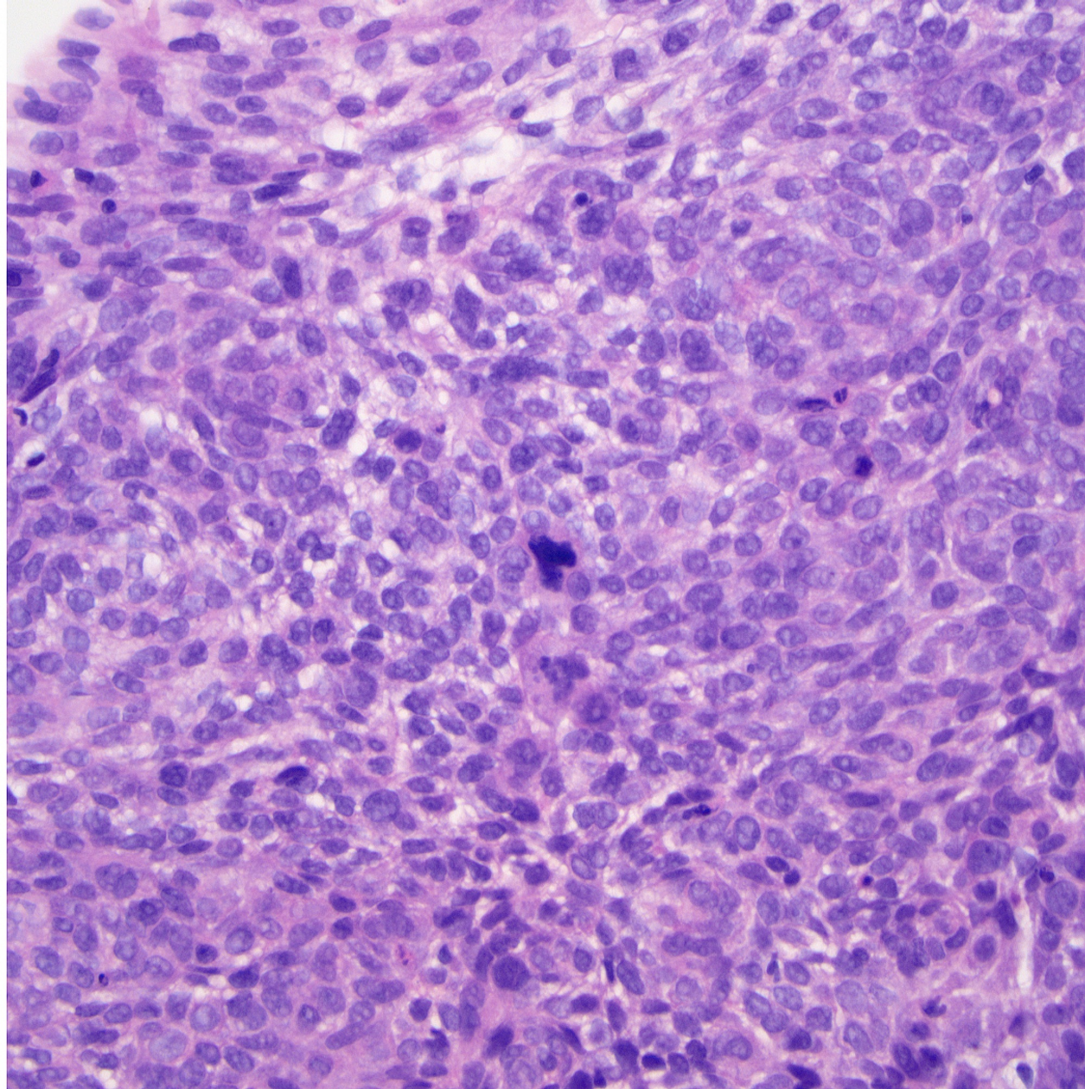
Biopsy from our patient’s esophagus showing basaloid morphology. There is a solid proliferation of densely packed cells with small, hyperchromatic nuclei, and scant cytoplasm. An atypical mitotic figure is present in the center of the image. H&E stain, 400x magnification. H&E: hematoxylin and eosin.

A 57-year-old African American female with a history of GERD and stroke was referred for two months of dysphagia to solids, unintentional weight loss of 30 lbs, and an abnormal barium swallow. The patient denied alcohol use and smoking. Barium swallow from the referring facility showed segmental narrowing and a long irregular distal esophageal stricture. Upper endoscopy revealed a circumferential ulcerated mass extending from 25 cm to 32 cm from the incisors, precluding passage of an adult gastroscope. The exchange for a pediatric gastroscope allowed for further evaluation and revealed a patch of nodular mucosa with ulceration at 23 cm from the incisors (Figure 1). In addition, a deep invasive, large bulky ulcerated fungating mass was seen (Figure 2). Pathology showed moderately to poorly differentiated basaloid SCC (Figure 3). The patient was referred to oncology and CT imaging was ordered for staging. Unfortunately, the patient also developed septic shock from which she eventually suffered a cardiac arrest, and thus CT imaging for staging was unable to be completed.

## Discussion

As in our case, patients with esophageal BSCC can present with non-specific symptoms of dysphagia, odynophagia, and weight loss. BSCC is predominantly seen in the upper aerodigestive tract (nasopharynx, tongue, larynx, and trachea), where it can be associated with human papillomavirus (HPV) [8]. In contrast, esophageal BSCC does not appear to be associated with HPV given the lack of positivity for high-risk HPV by in-situ hybridization [9]. Sarbia et al. noted that BSCC is frequently accompanied by conventional SCC [2]. Given this tumor heterogeneity, some cases that were originally diagnosed as conventional SCC on preoperative biopsy may later be re-classified as BSCC after the resected gross specimens reveal focal basaloid morphology in addition to conventional SCC [10]. Thus, sampling error and tumor heterogeneity can lead to diagnostic discrepancies between preoperative biopsies and resection specimens, which may complicate treatment decisions and research studies.

**Figure 4:**
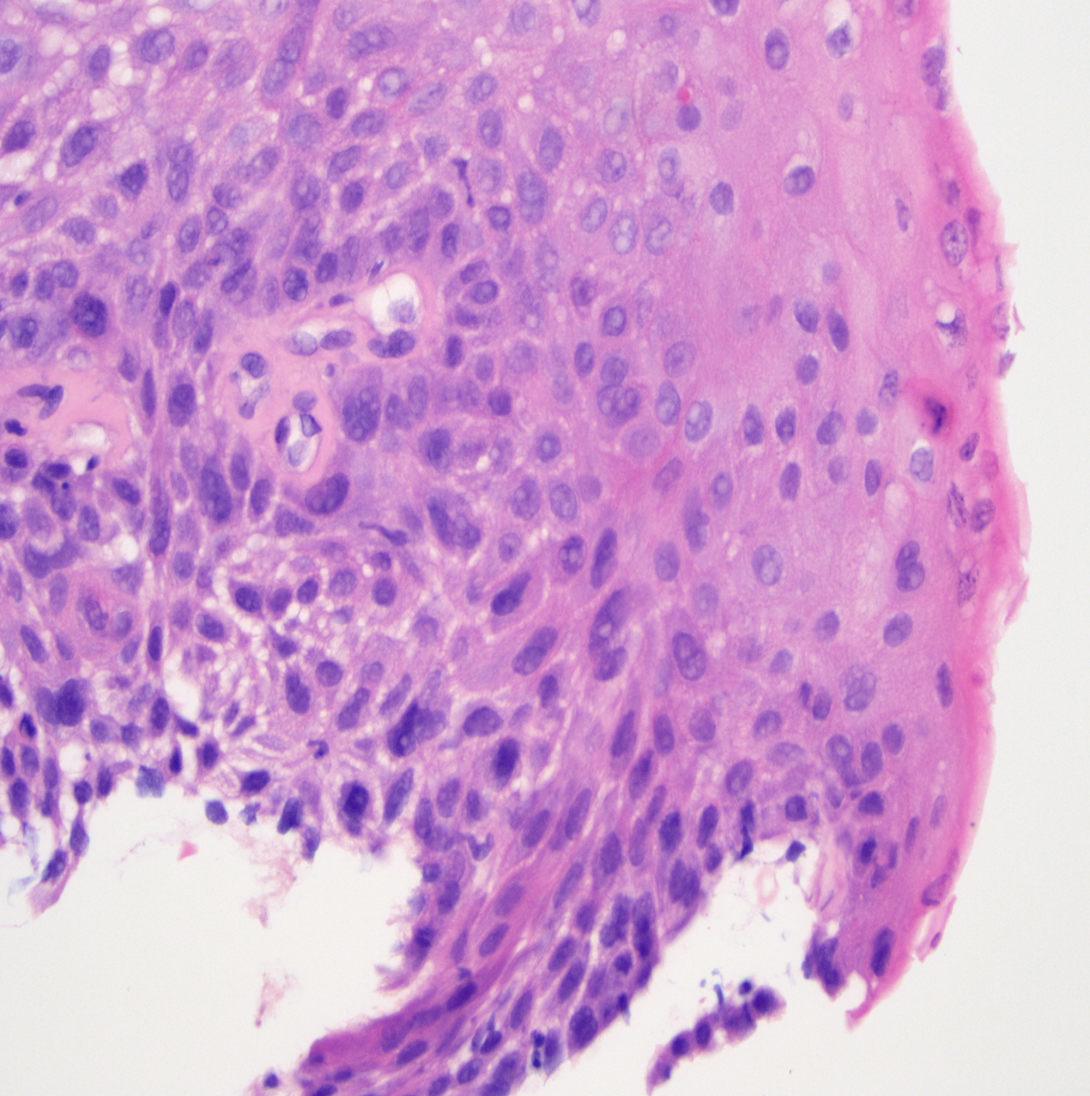
Biopsy from our patient’s esophagus showing focal areas of conventional SCC, which has preserved evidence of squamous differentiation. These areas have increased amounts of eosinophilic cytoplasm in the surface keratinocytes, which is seen on the right-hand side of the image. H&E stain, 400x magnification. SCC: squamous cell carcinoma, H&E: hematoxylin and Eosin.

Histologically, BSCC consists of small basaloid cells with oval to round nuclei, pale chromatin, and scant basophilic cytoplasm. The cells are arranged in solid or cribriform lobules with frequent comedo-necrosis [11]. However, there is no defined cutoff as to how much of the tumor should display this morphology. The main differential diagnoses to be excluded are high-grade neuroendocrine carcinomas, especially small cell carcinoma, and other basaloid neoplasms, such as adenoid cystic carcinoma. There is limited utility for immunohistochemical studies in this differential, as there is overlap in expression patterns between these entities. For example, markers of neuroendocrine differentiation (chromogranin A, synaptophysin, neuron-specific enolase, and Leu 7) typically positive in small cell carcinomas, and markers such as S100 and smooth muscle actin (typically positive in adenoid cystic carcinoma) may also show positivity in some BSCC. In addition, antibodies typical of squamous differentiation such as cytokeratins 13 and 14 show poor reactivity in BSCC. Furthermore, cytokeratin 14 may be expressed in adenoid cystic carcinomas with a similar frequency as BSCC [2]. However, immunohistochemical stains are not usually necessary for diagnosis as frequently there is admixed conventional SCC, focal squamous differentiation, or severe squamous dysplasia/carcinoma in situ in the adjacent epithelium that helps to confirm the squamous origin of the tumor [2]. Areas of conventional SCC were also seen in our patient’s biopsy material (Figure 4).

Morphologically, esophageal BSCC often shows higher proliferative activity and higher apoptotic indices when compared to typical squamous cell cancer of the esophagus [2]. Given these features, BSCC would appear to have a propensity for more aggressive behavior. BSCC has also been shown to have a high frequency of lymphovascular invasion and recurrence after resection, which also suggests poor prognosis [12]. However, several studies have shown that despite these worrisome features, the overall prognosis of patients with esophageal BSCC is similar to that of patients with typical SCC, although many studies are limited by small sample sizes. According to a retrospective study by Kumagai et al., there was no statically significant difference in the five-year survival rate (40% and 53%, respectively) when comparing nine cases of esophageal BSCC compared to 18 cases of typical SCC treated surgically [12]. In another study of 26 cases of BSCC by Chen et al. BSCC showed a statistically significant lower median survival time (MST) compared to well-differentiated SCC, but a similar MST when compared to moderately or poorly differentiated SCC [13]. Finally, in a study of 17 BSCCs and 133 typical SCCs conducted by Sarbia et al., no differences were found with regard to pT classiﬁcation, pN classiﬁcation, tumor size, blood vessel invasion, lymphatic vessel invasion, neural invasion, patient gender, or overall survival rates [2].

Due to its rare occurrence and lack of clear diagnostic criteria, it is challenging to study BSCC in the esophagus. Esophageal BSCC often co-exists with the conventional SCC, thus defining the proportion of the BSCC component required for classification may help to clarify the unique characteristics of this tumor, its clinico-pathologic tendencies, and responses to different therapeutic approaches. Currently, most BSCC are recommended to undergo surgical resection. However, recent studies suggest that BSCC might be highly sensitive to radiotherapy. There is a case report of an 86-year-old male with advanced BSCC who underwent radiotherapy alone with no recurrence for two years at the time the study was published in 2017 [14]. Maebayashi et al. also compared nine case reports of patients with advanced esophageal BSCC; resection was performed in all nine cases with three of those nine also receiving radiotherapy additionally. Two of the three who had received radiotherapy with surgery had a longer survival time than the median survival time of 13 months in the group [14]. There’s also another case report of a 59-year old male with esophageal BSCC who underwent endoscopic resection followed by radiation and the patient remained in remission without recurrence for 35 months when the study was published in 2018 [15]. Although these are mostly case reports with limited information, future studies comparing the efficacy of multimodal treatment including radiotherapy, chemotherapy, and surgery are warranted to establish a treatment algorithm.

## Conclusions

In conclusion, esophageal BSCC is a rare variant of SCC with the reported incidence ranging from 0.068% to 11%. BSCC has higher proliferative activity and apoptotic indices compared to the conventional SCC of the esophagus, which would suggest a propensity towards more aggressive behavior. However, several studies have shown that the prognosis of esophageal BSCC is similar to that of typical SCC without the basaloid component. Surgical resection remains the preferred treatment, but recent studies have shown that BSCC may be highly sensitive to radiotherapy. However, more studies are warranted to help establish its diagnostic criteria, clinical characteristics, and standard treatment.
